# Phylogenetic evidence of HIV-1 transmission linkage between two men who have sex with men

**DOI:** 10.1186/s12985-021-01573-5

**Published:** 2021-05-31

**Authors:** Jiafeng Zhang, Qin Fan, Mingyu Luo, Jiaming Yao, Xiaohong Pan, Xingguang Li

**Affiliations:** 1 Department of HIV/AIDS & STD Control and Prevention, Zhejiang Provincial Center for Disease Control and Prevention, No. 3399, Binsheng Road, Hangzhou, 310051 Zhejiang China; 2 Department of Technology R&D, Ningbo Institute of Life and Health Industry, University of Chinese Academy of Sciences, Ningbo, 315000 Zhejiang China

**Keywords:** HIV-1, CRF01_AE, Men who have sex with men, Phylogenetic analysis, Paraphyletic-monophyletic topology

## Abstract

**Background:**

In China, an HIV-infected man (complainant; P2) alleged that another man (defendant; P1) had unlawfully infected him with HIV through unprotected homosexual contact in 2018.

**Methods:**

We employed epidemiological, serological and phylogenetic analyses to investigate the transmission linkage between two men who have sex with men (MSM). Partial segments of three HIV-1 gene regions (*gag*, *pol*, and *env*) were amplified and sequenced by cloning. Maximum-likelihood (ML) and Bayesian methods were used to determine the direction and estimate the timing of transmission. Local control sequences and database control sequences were also used in the phylogenetic analysis.

**Results:**

It indicated that P2 underwent HIV seroconversion after P1 was diagnosed as HIV positive. The time to the most recent common ancestor (tMRCA) estimates consistently showed that P1 most likely became HIV-1 infected at an earlier date than P2. P1 and P2 were infected with the same HIV-1 CRF01_AE subtype according to segments of all three gene regions (*gag*, *pol*, and *env*). All three genetic regions of P1 have been subject to more potential selective forces than those of P2, indicating a longer evolutionary history. Bayesian and ML trees showed similar paraphyletic-monophyletic topologies of *gag* and *env*, with the virus from P1 located at the root, which supported a P1-to-P2 transmission direction.

**Conclusions:**

Phylogenetic investigations can elucidate HIV transmission linkage and might empower its use in the opposition of the intentional transmission of HIV-1 as a forensic tool.

## Background

Men who have sex with men (MSM) have become the core population of the HIV/AIDS epidemic around the world, including in China [1]. MSM are at increasing risk of HIV-1 infection due to engaging in anal intercourse, having multiple sex partners and showing low rates of condom use [1–3]. In China, a high prevalence of temporary sexual behavior has been reported among MSM [1], in which some HIV-infected individuals conceal their infection status, putting their sexual partner at a high risk of infection.

Since the “Florida Dentist case” was reported in the 1990s [4], phylogenetic analysis has been recurrently used as a forensic tool in HIV transmission investigations, including cases of nosocomial transmission [4, 5], outbreaks in prisons, father-to-child transmission [6], and sexual abuse [7, 8]. However, the forensic use of phylogenetic analysis to investigate the intentional transmission of HIV among MSM is scarce and is almost completely absent in China. Sanger sequencing has been used to obtain a consensus sequence representing the predominant viral strain (more than 20%) in a sample. HIV-1 is characterized by high genetic variability, due mainly to high rates of replication, mutation, and recombination. Transmitted variants may disappear or become minor variants of a viral quasispecies over time after transmission. Next-generation sequencing (NGS), molecular cloning/sequencing, and single-genome amplification (SGA)/sequencing are techniques that have been applied to study viral quasispecies [9], among which molecular cloning/sequencing is cost effective and easy to operate. Thus, phylogenetic analysis of the sequences of viral quasispecies provides a broad perspective regarding viral diversity and genetic evolution in an individual, which is important in the forensic context.

In the present study, we report a phylogenetic analysis of the sequences of viral quasispecies associated with alleged HIV-1 transmission between two HIV-infected MSM in China. In this case, an HIV-infected man (complainant; designated P2) alleged that another man (defendant; designated P1) had unlawfully infected him with HIV. The present study could promote the opposition of the intentional transmission of HIV-1 in China and beyond and provide molecular evidence for use in legal trials.

## Methods

### Subjects

P1 (defendant) was diagnosed with HIV-1 infection on March 29, 2018, when he was 22 years old. P2 (complainant) was 21 years old when he was diagnosed as HIV positive on June 14, 2018, by a municipal HIV-testing institution in Zhejiang Province (Zhoushan Municipal Center for Disease Control and Prevention, ZSCDC). P2 alleged that P1 had unlawfully infected him with HIV-1 via unprotected sexual contact without disclosing his HIV-positive status. Thereafter, a retrospective epidemiological investigation was carried out by the corresponding CDCs responsible for case follow-up. CDC staff administered a questionnaire to obtain demographic and risk behavior information after an HIV diagnosis. The questionnaire included demographic characteristics (age, gender, current residence, registered residence, migration), risk behavior characteristics (location, time range, condom use), characteristics of sexual partners (gender, number, methods of seeking sexual partners), and HIV testing history. Medical records related to HIV testing (e.g., HIV testing during invasive examination) were also collected.

Fresh anticoagulated whole-blood samples were collected from P1 and P2 under the supervision of police at the East Railway Station in Hangzhou on September 20, 2018 and September 21, 2018, respectively. P2 started ART (antiretroviral therapy) on June 28, 2018, while P1 did not receive ART at the time of sampling. Plasma samples from P1 and P2 collected within a month after diagnosis are available in the HIV-positive sample bank of the Zhejiang Provincial Center for Disease Control and Prevention (ZJCDC).

### Amplification, cloning, and sequencing

Genomic DNA was extracted from whole blood using the QIAamp DNA Blood Mini Kit (Qiagen, Valencia, CA, United States) following the manufacturer’s instructions. The PCR amplification of segments of the *gag* (HXB2: 781-1861, encoding part of p17 and p24), *pol* (HXB2: 2147-3462, encoding the protease gene and the first 300 codons of the reverse transcriptase gene) and *env* (HXB2: 7002-7541, encoding the V3-V4 region) gene regions of HIV-1, TA cloning, and sequencing were performed as previously described [10]. At least 20 clones per partial HIV-1 gene region were sequenced for each subject at each time point. Positive and negative controls were established through nucleic acid extraction, PCR amplification and molecular cloning. No nucleic acid cross-contamination occurred during the experiment.

### Phylogenetic analysis

The sequences were trimmed, assembled and adjusted with Sequencher v5.0 (Genecodes, Ann Arbor, MI). HIV subtyping using the Recombination Identification Program (RIP) tool (https://www.hiv.lanl.gov/content/sequence/RIP/RIP.html) from the Los Alamos National Laboratory (LANL) HIV Sequence Database (http://www.hiv.lanl.gov) indicated that the subtype of the query sequences of the segments of the *gag*, *pol*, and *env* gene regions of HIV-1 obtained from P1, P2, and P3 belonged to HIV-1 CRF01_AE. The reference sequences of subtypes A and CRF01_AE were retrieved from the LANL HIV Sequence Database (http://www.hiv.lanl.gov). For the database controls (DBCs), the sequences with the highest similarity were retrieved from the LANL HIV Sequence Database using the HIV BLAST tool (https://www.hiv.lanl.gov/content/sequence/BASIC_BLAST/basic_blast.html). Local control sequences of HIV-1 CRF01_AE circulating in the MSM population were randomly selected. The three final datasets of the segments of the *gag*, *pol*, and *env* gene regions of HIV-1 sequences were designated “dataset_gag”, “dataset_pol”, and “dataset_env”, respectively. Multiple sequence alignments of the three final datasets (“dataset_gag”, “dataset_pol”, and “dataset_env”) were performed using MAFFT v7.222 [11], and the alignment was subsequently manually edited using BioEdit v7.2.5. The best-fit nucleotide substitution models for the three final datasets were identified according to the Bayesian information criterion (BIC) method with three (24 candidate models) or 11 (88 candidate models) substitution schemes in jModelTest v2.1.10 [12].

Maximum-likelihood (ML) phylogenetic trees for the three final datasets were estimated using IQ-TREE v1.6.12 [13] under a Hasegawa–Kishino–Yano (HKY) [14] nucleotide substitution model with a gamma-distributed rate variation among sites (HKY + G) model, which was identified as the best fitting model for ML inference by jModelTest v2.1.10 [12]. Support for the inferred relationships was evaluated by bootstrap analysis with 1000 replicates. To assess the robustness of the ML tree topologies, we generated posterior probabilities for each node by performing Bayesian Markov Chain Monte Carlo (BMCMC) analyses implemented in MrBayes v3.2.7 [15].

### Reconstruction of time-scaled phylogenies

We applied a BMCMC method to estimate the time to the most recent common ancestor (tMRCA) for P1 and P2 at each time point, as implemented in BEAST v1.10.4 [16], and the BEAGLE v2.1.2 library program [17] was used for computational enhancement. To allow for variation in molecular evolutionary rates among lineages over time, we used the uncorrelated lognormal relaxed molecular clock with a discretized gamma-distributed general time-reversible substitution model. We ran Bayesian phylogenetic analyses using four coalescent tree priors (*i.e.*, constant size, expansion, exponential growth, and logistic). To ensure adequate mixing of the model parameters, MCMC chains were run for 100 million steps, with sampling every 10,000 steps from the posterior distribution. Convergence was evaluated by calculating the effective sample sizes of the parameters using Tracer v1.7.1 [18]. All parameters had an effective sample size > 200, which is indicative of sufficient sampling.

### Selective pressure analysis

Codon sites under selective pressure were analyzed in the segments of the *gag*, *pol*, and *env* gene regions of the HIV-1 sequences of P1 and P2 by using the HyPhy package [19] hosted on the Datamonkey web server [20]. Positively selected sites were detected using the single-likelihood ancestor counting (SLAC) [21], mixed-effects model of evolution (MEME) [22], fixed-effects likelihood (FEL) [21], and relaxed-effects likelihood (REL) [21] methods with statistical significance set at p < 0.1 (SLAC, MEME, and FEL) and a Bayes factor (BF) cutoff value of 50 (REL).

### Statistical analysis

Statistical analyses were conducted with SPSS v19.0 software (IBM, Armonk, NY). A* P *value < 0.05 was considered statistically significant.

## Results

### Epidemiological information and serological testing

**Fig. 1:**
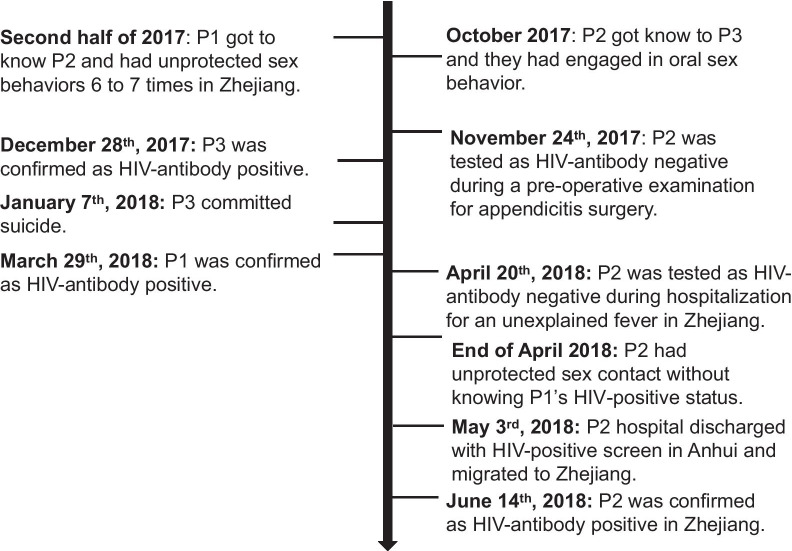
Timeline of HIV-1 transmission history among P1, P2 and P3. The timeline is not to scale

According to the detailed epidemiological investigation, P1 (defendant) was reported to be HIV-infected on March 29, 2018. At the end of April 2018, P2 alleged that P1 had unprotected sexual contact with him without disclosing his HIV-1 positive status. Thereafter, P2 was screened as shown to be HIV-1 positive by ELISA in a preoperative examination at Fuyang People’s Hospital of Anhui Province on May 3, 2018. P2 was diagnosed as HIV antibody indeterminate by the ZSCDC (Fig. 1), and the western blot results showed only four HIV-specific antibodies (anti-gp160, anti-p66, anti-p24, and anti-p17) on May 8, 2018. P2 was reported as HIV-1 positive, and western blot results identified gp160, gp120, p66, p55, p51, gp41, p31, p24, and p17 on June 14, 2018. Therefore, P2 underwent HIV seroconversion around May 2018, suggesting a recent infection.

Through contact tracing, we found that P2 had engaged in risky contact with P3. P2 alleged that he got to know P3 during the period from October to November 2017 and they had ever engaged in oral sex behaviors. P3 was confirmed as HIV-1 infected by the HIV confirmatory laboratory of ZSCDC on December 28, 2017. However, P3 committed suicide after his diagnosis. It was not possible to draw a subsequent blood sample from P3 to confirm the genetic relationship between P2 and P3. However, we obtained plasma samples from P3 after his diagnosis at the time of sampling on December 28, 2017 from the HIV-positive sample bank of the ZJCDC.

### Transmission linkage and direction

**Fig. 2:**
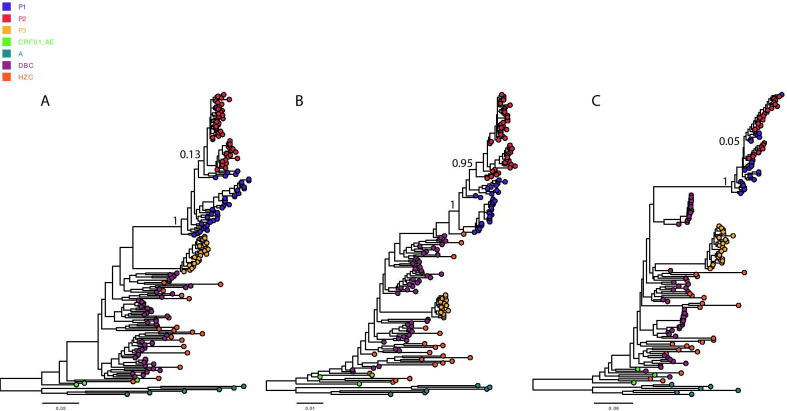
Bayesian tree reconstruction for *gag* (**a**), *pol *(**b**), and *env* (**c**) gene fragments. Viral sequences from the query sequences (P1, P2 and P3), local control sequences and database subtype CRF01_AE controls (DBCs) and CRF01_AE reference sequences are colored differently. Subtype A reference sequences, shown in dark cyan, were used as an outgroup for the rooting of phylogenetic trees. The Bayesian posterior probability (PP) values are indicated above the branches associated with the case sequences; DBC, database controls; HZC, local controls

**Fig. 3:**
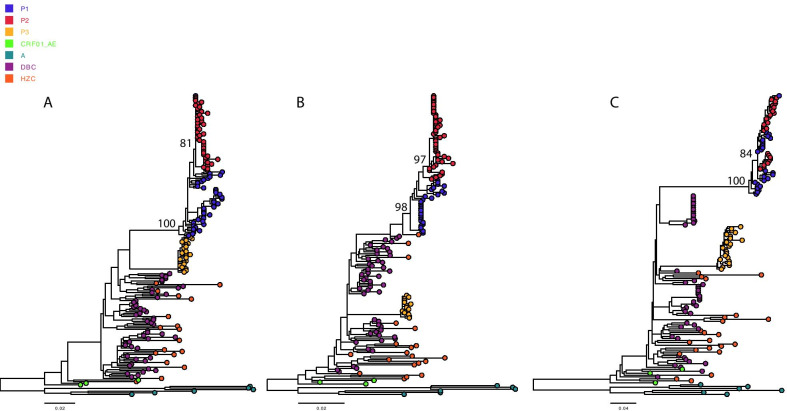
ML tree reconstruction for *gag* (**a**), *pol* (**b**), and *env* (**c**) gene fragments. Viral sequences from query sequences (P1, P2 and P3), local control sequences and database subtype CRF01_AE controls (DBCs) and CRF01_AE reference sequences are colored differently. Subtype A reference sequences, shown in dark cyan, were used as an outgroup for the rooting of phylogenetic trees. Node support values are indicated on the case sequences. ML, maximum-likelihood; DBC, database controls; HZC, local controls

Partial segments of the *gag*, *pol* and *env* gene regions of HIV-1 were amplified, TA cloned and bidirectionally sequenced. The HIV-1 quasispecies obtained from P1, P2, and P3 within the three gene regions of HIV-1 were identified by cloning and sequencing. According to Bayesian reconstruction, the three datasets (“dataset_gag”, “dataset_pol”, and “dataset_env”) of the sequences from P1 and P2 formed a strongly supported transmission cluster (Bayesian posterior probability = 1) within HIV-1 CRF01_AE. In “dataset_gag” and “dataset_env”, a subset of sequences from P2 were paraphyletic with respect to those of P1, showing a paraphyletic–monophyletic (PM) topological relationship in which P1 was inferred to be located at the root (Bayesian posterior probability = 1). In “dataset_pol”, the sequences from P1 and P2 displayed a monophyletic-monophyletic (MM) topological relationship (Fig. 2). Similar topological relationships were also inferred using the ML approach (Fig. 3). We inferred that HIV-1 transmission had occurred in a P1-to-P2 direction. The sequences obtained from P3 were clustered in a significantly separate cluster from the sequences obtained from P1 and P2. The phylogenetic analysis conducted in the present study excluded P3 as a source of HIV-1 transmission to P2.

### Transmission time estimation

tMRCA estimation consistently showed that P1 most likely became HIV-1 infected at an earlier date than P2 according to two of the analyzed gene fragments (*gag* and *pol*) (mean tMRCA *gag* P1 = December 2017, P2 = April 2018; *pol* P1 = December 2017, P2 = May 2018) (Table. 1) for most of the coalescent tree priors tested. Interestingly, P2’s tMRCA estimate agreed well with his symptoms of acute HIV-1 infection in May 2018.

**Table 1:** Bayesian phylogenetic tMRCA estimates for genomic regions of HIV-1 CRF01_AE from subjects P1 (defendant) and P2 (prosecutor) under coalescent tree priors

Genomic region	Coalescent tree prior	Subject P1 (defendant)			Subject P2 (prosecutor)		
		Mean	Lower 95% HPD	Upper 95% HPD	Mean	Lower 95% HPD	Upper 95% HPD
*gag*	Constant	2017-12-10	2017-07-25	2018-03-22	2018-04-28	2018-01-24	2018-06-18
	Expansion	2017-12-08	2017-07-24	2018-03-25	2018-04-27	2018-01-22	2018-06-18
	Exponential	2018-01-31	2017-11-14	2018-03-29	2018-05-01	2018-01-29	2018-06-18
	Logistic	2017-12-06	2017-07-21	2018-03-27	2018-04-26	2018-01-15	2018-06-18
*pol*	Constant	2017-12-02	2017-03-25	2018-04-07	2018-05-15	2018-03-22	2018-06-18
	Expansion	2017-12-23	2017-05-20	2018-04-05	2018-05-16	2018-03-25	2018-06-18
	Exponential	2018-02-07	2017-11-01	2018-04-04	2018-05-17	2018-03-30	2018-06-18
	Logistic	2017-12-13	2017-04-14	2018-04-06	2018-05-18	2018-04-04	2018-06-18

### Potential selection analysis

The segments of the *gag*, *pol*, and *env* gene regions of HIV-1 harbored more codon sites that were affected by potential selective forces in P1 than in P2. Notably, P1 also exhibited more codon sites affected by positive and negative selection in the segments of the *gag*, *pol*, and *env* gene regions of HIV-1 than did P2 (Table 2).

**Table 2:** Codon sites under selective pressure in the analyzed gene fragments for subjects P1 (defendant) and P2 (prosecutor)

Genomic region	Subject P1 (defendant)	Subject P2 (prosecutor)
*gag*	**82**, 10, 39, 124, 132, 133, 154, 159, 182, 201, 207, 233, 255, 263, 270, 304, 306, 309, 312, 313, 321	–
*pol*	**201**, 17, 197, 312, 323, 342, 350, 374	253, 290, 359, 376
*env*	**23**, **79**, **138**, 4*, 5*, 7, 75, 107, 172, 210*	**121**, **206**, 4*, 5*, 197, 210*

Bold figures and underlined figures in the table denote sites under positive and negative selective pressure, respectively. Same sites that are under selective pressure in both subjects are denoted with asterisks (*)

## Discussion

In the present study, we employed epidemiological, serological and phylogenetic analyses to investigate a potential case of HIV-1 transmission between two MSM. The defendant (P1) had unprotected sexual contact without disclosing his HIV-positive status with the complainant (P2), which was revealed by an epidemiological investigation (Fig. 1). The defendant (P1) was reported to be HIV-positive earlier than the complainant (P2). The complainant (P2) was recently infected, as indicated by the typical phenomenon of seropositive conversion according to the progression of WB bands. As shown in Table 1, the tMRCA estimation based on segments of the *gag* and *pol* gene regions of HIV-1 consistently showed that P1 most likely became HIV-1 infected at an earlier date than P2. The *gag*, *pol*, and *env* gene regions of HIV-1 were subjected to more potential selective forces in P1 than in P2, indicating a longer evolutionary history of the strain in P1 (Table 2). Furthermore, in the comparison of the local circulating strains and the most related sequences from the HIV database (https://www.hiv.lanl.gov/content/index), the viral sequences from the two individuals exhibited a high level of similarity and were most closely related to each other. Furthermore, a paraphyletic relationship was observed for the *gag* and *env* gene sequences of P1 and P2, suggesting that P1 was the source of infection from the point of view of molecular evidence. In conclusion, by combining epidemiological information and serological testing, the relatedness and transmission direction of the HIV-1 strains of the subjects were proven by phylogenetic analysis, supporting the epidemiologically identified linkage in this case.

Phylogenetic analysis results should be interpreted with caution. Because the complete sampling of all relevant individuals belonging to the same transmission event is not feasible, it cannot completely exclude the possibility of underlying intermediates [9, 23]. This situation is worth considering because MSM in China display a high level of sexual activity, including exhibiting multiple sex partners, especially casual sexual partners [24]. Phylogenetics can be used to exonerate individuals if the sequences of defendants cluster significantly separately from those of complainants [9, 23]. However, the longer the time since infection, the greater the diversity separating viruses infecting linked individuals will be [9, 25]. In the present study, P3 was suspected to be a potential source of infection by partner tracking. However, P3 showed clustering in a significantly separate cluster in the phylogenetic trees for all three genetic regions; thus, it is logical to exonerate P3. This finding is consistent with a supplementary epidemiological investigation indicating that P2 only engaged in oral sex with P3 without any anal sex behaviors according to the self-report of P2. The direction of transmission is an important and challenging issue in the context of forensic HIV transmission investigations [9, 25]. A phylogenetic analysis of transmission direction can be performed in the context of multiple clones (or samples) representing patients’ viral quasispecies [9, 25]. In this study, the viral quasispecies of P1 exhibited a paraphyletic relationship with those from P2 in the Bayesian phylogenetic trees of the segments of the *gag* and *env* gene regions of HIV-1 as well as in the ML tree. The results not only supported a high level of similarity between P1 and P2 but also supported a P1-to-P2 transmission direction. As reported previously, phylogenetics can be used to support the investigation of HIV transmission in the context of other types of evidence [9]. We determined the infection window period for P2 based on serological results, and the result was consistently supported by the tMRCA estimates obtained for P2 using molecular clock models.

Leitner et al. [9, 26] concluded that the accuracy of a reconstructed tree topology was more dependent on the amount of genetic information than on the phylogenetic reconstruction methods used. Most investigations involving HIV cases target *env*, sometimes combined with the analysis *gag* or *pol *[9]. In this study, we analyzed segments of the *gag, env* and *pol* gene regions of HIV-1 to generate more reliable results. We found that ML trees of the segments of the *gag* and *env* gene regions of HIV-1 showed a PM topology for P1 and P2. However, the ML tree of a segment of the *pol* gene region of HIV-1 showed an MM topology for P1 and P2. These results were consistent with the Bayesian analyses (Figs. 2 and 3). A paraphyletic relationship was not observed for the *pol* gene region, possibly due to the deviation of the cloning method because of the limited available clones. This illustrates the importance of evaluating more than one gene region, as there are unlikely to be identical sampling artifacts across several gene regions. Furthermore, we used different phylogenetic reconstruction methods (ML and Bayesian estimates) to verify the consistency of the obtained results. The results showed that the ML and Bayesian estimates were concordant, providing mutual support for the reliable results.

### Limitations

Due to loss to follow-up and lack of other types of evidence, it is still difficult to confidently estimate the infection window period for P1, although the tMRCA of P1 was estimated using molecular clock models. We failed to amplify the segment of the *env* gene region of HIV-1 in later samples from P1 and P2 despite many efforts; therefore, we could not estimate the tMRCA from the segment of the *env* gene region of HIV-1 by the Bayesian inference method. We found that P2’s tMRCA estimate was surprisingly consistent with the timing of his HIV seroconversion. A recent study showed that genetic diversity calculated from NGS data enables a more accurate estimation of coalescence times [27]. However, the reliability of such estimations requires further investigation and validation, especially in the context of court cases.

## Conclusions

In summary, the PM phylogeny, root host label, timing and selection analysis results all supported the occurrence of HIV transmission between the two MSM involved in this case. The molecular data strongly supported the epidemiological conclusion that P1 transmitted HIV-1 to P2.

## Data Availability

The datasets used and analyzed during the current study are available from the corresponding author on reasonable request.

## Abbreviations

AIDS: Acquired immune deficiency syndrome
ART: Antiretroviral therapy
BMCMC: Bayesian Markov Chain Monte Carlo
CDC: Center for Disease Control and Prevention
DBCs: Database controls
HIV: Human immunodeficiency virus
HKY: Hasegawa–Kishino–Yano
LANL: Los Alamos National Laboratory
ML: Maximum-likelihood
MM: Monophyletic-monophyletic
MSM: Men who have sex with men
NGS: Next-generation sequencing
PM: Paraphyletic–monophyletic
tMRCA: Time to the most recent common ancestor
